# Imaging biomarkers for early detection, monitoring, and prediction of treatment- related side effects after radiotherapy: A scoping review

**DOI:** 10.21203/rs.3.rs-11296636/v1

**Published:** 2026-10-18

**Authors:** Natalie A. West, Eliana Gioscio, Azadeh Abravan, Jane Shortall, Giulia Rambaldi Guidasci, Vanessa Panettieri, David P. Farris, Peter van Luijk, Claudio Fiorino, Robert Jeraj, Tiziana Rancati, Clifton D. Fuller, Laia Humbert-Vidan

## Abstract

Background/Purpose Imaging biomarkers can be used in radiotherapy for prediction of radiation-induced injury, monitoring tumor response, and risk modeling. Despite their clinical potential, imaging biomarkers have not yet achieved large-scale clinical adoption. The purpose of this scoping review was to assess existing studies related to imaging biomarkers for radiotherapy-induced side effects and evaluate the disconnect between translational research and clinical implementation. Methods Systematic searches of MEDLINE, Embase, and Cochrane Library (inception-January 2025) yielded 2,733 unique records after deduplication. Independent dual-reviewer screening and data extraction were conducted in Covidence, with consensus resolution for disagreements. Extracted variables included study design, cancer type, imaging modality, validation approach, clinical endpoint, BEST biomarker category, clinical implementation status, and regulatory approval. Results Of 2,733 screened records, 102 studies (2007–2024) were included. Neurologic and head and neck cancers comprised 67% of studies. Predictive biomarkers were most common (53%), followed by diagnostic (25%), prognostic (22%), and monitoring (18%). Most studies were single-institution (85%), and 57% did not technically validate their biomarkers; external validation occurred in only 6%. No studies reported clinical implementation or regulatory approval. Conclusion Despite growing research interest, imaging biomarkers for radiotherapy-related normal tissue damage remain almost entirely investigational, with minimal clinical implementation and regulatory approval. Bridging this translational gap requires rigorous external validation, methodological standardization, and structured pathways toward regulatory qualification.

### 1. Introduction

Radiotherapy plays a central role in the treatment of cancer, either alone or in combination with surgery and/or systemic therapies in 50%-75%[1–4] of patients. Advances in treatment planning, image guidance, and delivery techniques have improved the therapeutic ratio and reduced exposure of normal tissues to radiation. Nevertheless, radiation-induced normal tissue damage remains a major clinical challenge. Acute and late side effects may result in substantial morbidity, impaired functional outcomes, reduced quality of life, and increased healthcare utilization[5–7]. As the population of cancer survivors continues to grow, the importance of minimizing radiation-induced side effects and identifying tissue injury at a stage when interventions may still be effective is increasing.

Many side effects are diagnosed only after clinically apparent symptoms have developed, potentially limiting opportunities for early intervention. The lack of objective, standardized, and sensitive measures of tissue injury may contribute to heterogeneous diagnosis, monitoring, management, and reporting of radiation-induced side effects across institutions. On the other hand, radiation-induced side effects are predominantly assessed using subjective approaches. Normal tissue damage is commonly evaluated through clinician-reported grading systems, such as the Common Terminology Criteria for Adverse Events (CTCAE)[8], alongside patient-reported outcome measures (PROMs)[9]. While these instruments are clinically valuable and standardized, they remain inherently subjective and are often influenced by inter-observer variability, reporting inconsistencies, and differences in clinical practice. Imaging biomarkers offer considerable potential to address these limitations. Modern imaging techniques can provide quantitative information on structural, functional, metabolic, and physiological tissue changes that may precede the development of clinically detectable side effects. Imaging biomarkers have therefore been investigated for a range of applications including early detection of radiation-induced injury, longitudinal monitoring of tissue response, risk stratification, treatment adaptation, and prediction of future damage[10]. Compared with conventional clinical assessments, imaging biomarkers may provide more objective, reproducible, and standardized measures of tissue injury, while potentially also enabling earlier identification of patients at increased risk of adverse outcomes. Consequently, they have emerged as a promising component of precision radiation oncology[11].

However, despite growing interest and ongoing research efforts, clinical implementation of imaging biomarkers for radiation damage remains limited. Many proposed biomarkers have been developed in small single-center cohorts, lack external validation, and are affected by variability in image acquisition, processing, segmentation, feature extraction, and reporting. Additional challenges include insufficient technical and clinical validation, limited demonstration of clinical utility, uncertainty regarding regulatory pathways, and poor integration into existing clinical workflows. Recognition of these barriers has led to the publication of multiple methodological frameworks, reporting guidelines, standardization initiatives, and implementation roadmaps aimed at improving the development, validation, and translation of imaging biomarkers into clinical practice[12,13].

Technical standardization efforts, such as the Quantitative Imaging Biomarkers Alliance (QIBA)[14,15] and the Image Biomarker Standardization Initiative (IBSI)[16], have aimed to improve reproducibility and comparability across imaging platforms, acquisition protocols, and analytical pipelines. In parallel, reporting and quality assessment frameworks including the Radiomics Quality Score (RQS)[17,18], TRIPOD(-AI)[19], STARD[20], CLAIM[21], REMARK[22], CLEAR[23], and METRICS[24] have provided guidance on study design, validation, transparency, and reporting. Broader imaging biomarker roadmaps developed by organizations such as the European Society of Radiology (ESR), the European Imaging Biomarkers Alliance (EIBALL), and related international working groups have further highlighted the critical steps required for successful clinical translation, including technical validation, biological validation, clinical validation, demonstration of clinical utility, regulatory approval, and integration into healthcare workflows[25]. The FDA-NIH BEST (Biomarkers, EndpointS, and other Tools) framework has additionally provided a common terminology and classification system for biomarker development and application[26]. These initiatives provide a framework for the development of robust and clinically relevant imaging biomarkers. However, the extent to which the radiation-related side effect imaging biomarker literature has adopted these recommendations and progressed towards routine clinical implementation remains unclear.

Therefore, there is a need to evaluate the current state of imaging biomarker research in radiation-related side effects through the lens of these established translational frameworks. This scoping review aims to characterize the current landscape of imaging biomarkers developed for the detection, monitoring, and prediction of radiation-induced side effects across cancer types and treatment modalities. Specifically, we sought to identify the imaging biomarkers being investigated, assess the extent of their technical and clinical validation, evaluate evidence of clinical implementation and translation, and examine adherence to existing recommendations for biomarker development, standardization, reporting, and validation. By mapping the current evidence base and identifying persistent methodological and translational gaps, this review aims to inform future research priorities and support the development of imaging biomarkers that are capable of achieving meaningful clinical impact.

### 2. Materials and Methods

## 2.1 Database search

This scoping review followed PRISMA-ScR guidelines (Supplementary Table S5) and had a protocol registered in OSF; DOI: 10.17605/OSF.IO/CVMUJ)[27]. A medical research librarian (DPF) searched MEDLINE (Ovid), Embase (Ovid), and Cochrane Library (Wiley) from inception to January 22, 2025, with the searches executed on January 23, 2025. After consultation with the research team, the librarian selected controlled vocabulary (MeSH and Emtree) and natural language terms for the concepts of imaging biomarkers and radiation-related side effects. The search strategy was customized for each database, and no limiters were used (Supplementary Table S1).

## 2.2 Screening and data extraction

Screening and data extraction were performed independently by at least two reviewers in each step using Covidence Systematic Review Software (Veritas Health Innovation, Melbourne, Australia)[28], with disagreements resolved by a third independent reviewer and, when necessary, through discussion and consensus. The search strategy for each database is available in the supplementary materials. Eligibility criteria were defined a priori using the PICO framework and are reported in full in the supplementary materials (Supplementary Table S2). A standardized data extraction template was developed a priori and piloted by the review team. Extracted variables encompassed study characteristics, population and treatment details, imaging modalities and biomarkers, technical validation procedures, associations with clinical and dosimetric outcomes, modeling approaches, validation methods, clinical implementation, regulatory considerations, and key study findings. The template was designed not only to characterize the existing evidence base but also to capture elements relevant to biomarker development, validation, reporting quality, and clinical translation. The full data extraction template is provided in Supplementary Table S3.

## 2.3 Analysis

Data was extracted from Covidence as a CSV file. Data cleaning and analysis were performed in JMP (Student Edition, v18.2.2). Variables including “Region Study was Conducted,” “Type of Study,” “Study Design,” “Study Population Size,” “Gender Distribution,” “Clinical Endpoint,” “Clinical Endpoint Assessment,” “Treatment Modalities,” “Imaging Modalities Used,” “ML/DL Methods,” and “Model Performance” were recoded for clarity and to remove outliers. Cancer type was recoded to align with the Cancer Types by System as defined by the American Cancer Society[29]. BEST Biomarker Category was reviewed by a single reviewer for accuracy and consistency[26].

### 3. Results

## 3.1 Search results

A total of 3,503 results were retrieved from the three databases including the set of 2 key articles supplied by the research team (MEDLINE = 843; Embase = 2185; Cochrane = 475). Search results were uploaded to Covidence, and after deduplication, 2,727 unique records remained for title and abstract screening. After full-text review, 102 studies met the eligibility criteria and were included in the final analysis. The study selection process and reasons for exclusion at each stage are presented in the PRISMA flow diagram (Fig. 1).

## 3.2 Data Extraction Results

## 3.2.1 Overview

Table 1 presents an overview of the extracted data from the 102 included studies in this review. Supplementary Table S4 contains additional information on these studies. Studies were published between 2007 and 2024, with 82% of studies published within 2016–2024 (Fig. 2). 43% of studies were conducted in North America, followed by 30% in Asia-Pacific, 22% in Europe, and 5% in the Middle East. 84% of studies included a clear inclusion/exclusion workflow, specifying the criterion used in the final cohort. 85% of studies were conducted at a single institution and no studies included an Enhancing the QUAlity and Transparency Of health Research (EQUATOR) checklist[30].

Table 1

**Comprehensive listing of final studies analysed in this scoping review ordered by year of publication.** Additional details are included in Sup

| Study ID | Region | Type | Timing | Temp. | Clin. Impl. | Study Val. | Cancer | Clin. Endpoint | BEST Cat. | Imaging |
|---|---|---|---|---|---|---|---|---|---|---|
| Bishay 2024[31] | N. America | Clinical (RWD) | Retrosp. | Long. | No | Int. | H&N | Cognitive function | Monitoring | PET-CT |
| Choi 2023[32] | N. America | Clinical (RWD) | Retrosp. | Cross sect. | No | Int. & Ext. | Thoracic | Cardiac injury | Predictive; Prognostic | PET-CT |
| Delasos 2024[33] | N. America | Clinical (RWD) | Retrosp. | Long. | No | Int. & Ext. | Thoracic | Pneumonitis | Diagnostic | CT |
| Heyn 2025[34] | N. America | Clinical (RWD) | Retrosp. | Cross sect. | No | None | Neuro. | Brain necrosis | Diagnostic; Predictive; Pharmacodynamics or response | MRI |
| Khalaj 2024[35] | N. America | Clinical (RWD) | Retrosp. | Long. | No | None | Neuro. | Brain necrosis | Diagnostic; Monitoring; Predictive | MRI |
| Klaar 2024[36] | Europe | Clinical (RWD) | Prosp. | Long. | No | Int. | Thoracic | Pneumonitis | Predictive | CT; MRI |
| Li 2024[37] | Asia-Pacific | Clinical (RWD) | Retrosp. | Cross sect. | No | None | H&N | Brain injury; Cognitive function | Monitoring | MRI |
| Lorimer 2024[38] | Europe | Clinical (RWD) | Prosp. | Long. | No | None | Neuro. | Brain injury; Cognitive function | Predictive; Prognostic | MRI |
| Mao 2024[39] | N. America | Clinical (RWD) | Retrosp. | Long. | No | None | H&N | Dysphagia; Fibrosis | Predictive | MRI |
| Piffer 2024[40] | Europe | Clinical (RWD) | Retrosp. | Cross sect. | No | Int. | Neuro. | Brain injury; Cognitive function | Predictive | MRI |
| Sadique 2024[41] | N. America | Clinical (RWD) | Retrosp. | Cross sect. | No | Int. | Neuro. | Brain necrosis | Prognostic | MRI |
| Schack 2024[42] | Europe | Clinical (RWD) | Retrosp. | Cross sect. | No | None | Neuro. | Brain necrosis | Diagnostic; Prognostic | MRI |
| Yang 2024[43] | Asia-Pacific | Clinical (RWD) | Retrosp. | Cross sect. | No | Int. | GI | Gastrointestinal injury | Predictive | CT |
| Zheng 2023[44] | Asia-Pacific | Clinical (RWD) | Prosp. | Cross sect. | No | None | H&N | Brain necrosis | Diagnostic | MRI |
| Alirezaei 2023[45] | Middle East | Clinical (RWD) | Prosp. | Long. | No | Int. | Neuro. | Brain necrosis | Predictive; Prognostic | MRI |
| Bergamaschi 2023[46] | Europe | Clinical (RWD) | Retrosp. | Cross sect. | No | None | H&N | Sarcopenia | Safety | CT |
| Lin 2023[47] | Asia-Pacific | Clinical (RWD) | Prosp. | Long. | No | Int. | H&N | Necrosis | Monitoring; Predictive; Prognostic | MRI |
| Liu 2023[48] | Asia-Pacific | Clinical (RWD) | Retrosp. | Cross sect. | No | Int. & Ext. | H&N | Necrosis | Predictive; Prognostic | MRI |
| McSweeney 2023[49] | Europe | Clinical (RWD) | Retrosp. | Cross sect. | No | Int. | GI | Lymphopenia | Prognostic | CT |
| Trada 2023[50] | Asia-Pacific | Clinical (RWD) | Prosp. | Long. | No | Int. | H&N | Xerostomia | Predictive | PET-CT |
| Bao 2022[51] | Asia-Pacific | Clinical (RWD) | Retrosp. | Cross sect. | No | Int. | H&N | Brain injury | Prognostic | MRI |
| Fanizzi 2022[52] | Europe | Clinical (RWD) | Retrosp. | Cross sect. | No | Other | H&N | Xerostomia | Predictive; Prognostic | CT |
| Lin 2022[53] | Asia-Pacific | Clinical (RWD) | Retrosp. | Long. | No | Ext. | H&N | Brain necrosis | Predictive | MRI |
| Pruijssen 2022[54] | Europe | Clinical (RWD) | Prosp. | Cross sect. | No | None | H&N | Vasculopathy | Predictive | MRI; Ultrasound |
| Qin 2022[55] | Asia-Pacific | Clinical (RWD) | Retrosp. | Cross sect. | No | Int. | H&N | Brain injury; Cognitive | Diagnostic | MRI |

| Study ID | Region | Type | Timing | Temp. | Clin. Impl. | Study Val. | Cancer | Clin. Endpoint | BEST Cat. | Imaging |
|---|---|---|---|---|---|---|---|---|---|---|
| | | | | | | | | function | | |
| Speers 2022[56] | N. America | CT | Prosp. | Long. | No | Int. | Breast | Cardiac injury | Predictive | MRI |
| SvanstromRojvall 2022[57] | Europe | CT | Prosp. | Long. | No | None | GI | Bone injury | Predictive; Pharmacodynamics or response | MRI |
| Thomas 2022[58] | N. America | Clinical (RWD) | Prosp. | Long. | No | None | Thoracic | Pneumonitis | Prognostic | 4DCT; SPECT; CT |
| Alirezaei 2021[59] | Middle East | Clinical (RWD) | Prosp. | Long. | No | None | Neuro. | Brain injury; Cognitive function | Predictive | MRI |
| Bontempi 2021[60] | Europe | Clinical (RWD) | Prosp. | Long. | No | None | Neuro. | Brain injury | Diagnostic | MRI |
| Beukema 2022[61] | Europe | Clinical (RWD) | Prosp. | Cross sect. | No | Int. & Ext. | GI | Fibrosis | Diagnostic | CT; Echocardiograph MRI |
| Bousabarah 2021[62] | Europe | Clinical (RWD) | Retrosp. | Long. | No | Ext. | Thoracic | Fibrosis | Predictive | CT |
| Elhalawani 2021[63] | N. America | Clinical (RWD) | Retrosp. | Long. | No | None | H&N | Xerostomia | Predictive | PET-CT |
| Go 2021[64] | Asia-Pacific | Clinical (RWD) | Retrosp. | Long. | No | None | Thoracic | Cachexia; Hematological injury | Monitoring | CT |
| Gommlich 2022[65] | Europe | Clinical (RWD) | Prosp. | Long. | No | Int. | Neuro. | Brain necrosis; Oedema | Diagnostic; Prognostic | MRI |
| Hu 2022[66] | Asia-Pacific | CT | Retrosp. | Long. | No | None | Neuro. | Brain injury | Prognostic | MRI |
| Jahangiri 2021[67] | N. America | Clinical (RWD) | Prosp. | Long. | No | Int. | Thoracic | Lung injury | Predictive | PET; PET-CT |
| Lin 2021[68] | Asia-Pacific | Clinical (RWD) | Retrosp. | Long. | No | None | H&N | Brain injury; Cognitive function | Monitoring; Predictive | MRI |
| Marzi 2021[69] | Europe | Clinical (RWD) | Prosp. | Long. | No | Int. | H&N | Xerostomia | Predictive; Pharmacodynamics or response | CT; MRI |
| Matsuo 2021[70] | Asia-Pacific | CT | Prosp. | Long. | No | None | Thoracic | Sarcopenia; Systematic inflammation | Prognostic | CT |
| Otman 2021[71] | Europe | Clinical (RWD) | Retrosp. | Cross sect. | No | None | Neuro. | Brain necrosis | Prognostic | MRI; PET-CT |
| Smyczynska 2021[72] | Europe | Clinical (RWD) | Retrosp. | Long. | No | Int. | H&N | Hypothyroidism | Predictive | CT |
| Burke 2020[73] | N. America | Clinical (RWD) | Prosp. | Long. | Yes | None | GI | Cardiac injury; Ischemia | Safety | MRI |
| Hirose 2020[74] | Asia-Pacific | Clinical (RWD) | Retrosp. | Cross sect. | No | Int. | Thoracic | Pneumonitis | Predictive | CT |
| Huynh-Le 2021[75] | N. America | CT | Prosp. | Long. | No | None | Neuro. | Brain injury | Prognostic | MRI |
| Qin 2020[76] | Asia-Pacific | Clinical (RWD) | Retrosp. | Long. | No | None | Thoracic | Pneumonitis | Predictive | CBCT; CT |
| Tibbs 2020[77] | N. America | CT | Prosp. | Long. | No | None | Neuro. | Brain injury | Predictive | MRI |
| Wilkie 2020[78] | N. America | Clinical (RWD) | Retrosp. | Cross sect. | No | Int. & Ext. | H&N | Xerostomia | Predictive | PET-CT |

| Study ID | Region | Type | Timing | Temp. | Clin. Impl. | Study Val. | Cancer | Clin. Endpoint | BEST Cat. | Imaging |
|---|---|---|---|---|---|---|---|---|---|---|
| Abdollahi 2019[79] | Middle East | Clinical (RWD) | Prosp. | Long. | No | None | GU | Bone injury | Diagnostic | MRI |
| Abdollahi 2019[80] | Middle East | Clinical (RWD) | Prosp. | Long. | No | None | GU | Cystitis | Monitoring; Predictive | MRI |
| Bian 2019[81] | Asia-Pacific | Clinical (RWD) | Prosp. | Long. | No | Int. | Neuro. | Cognitive function | Predictive | MRI |
| Chen 2019[82] | N. America | Clinical (RWD) | Prosp. | Long. | No | Int. | Neuro. | Brain injury | Diagnostic | MRI |
| Leng 2019[83] | Asia-Pacific | CT | Prosp. | Long. | No | None | H&N | Brain injury | Diagnostic | MRI |
| Richter 2019[84] | N. America | CT | Prosp. | Long. | No | None | GI | Liver injury | Predictive | MRI |
| Saednia 2020[85] | N. America | Clinical (RWD) | Prosp. | Long. | No | Int. | Breast | Skin injury | Predictive | Thermal Imaging |
| Sun 2019[86] | Asia-Pacific | Clinical (RWD) | Prosp. | Long. | No | None | GI | Cognitive function | Predictive | MRI |
| Tringale 2019[87] | N. America | Clinical (RWD) | Prosp. | Long. | No | None | Neuro. | Cognitive function | Predictive; Pharmacodynamics or response | MRI |
| Beshr 2017[88] | Asia-Pacific | Clinical (RWD) | Retrosp. | Long. | No | Int. | Neuro. | Brain necrosis | Diagnostic | PET-CT |
| Ding 2018[89] | Asia-Pacific | Clinical (RWD) | Prosp. | Cross sect. | No | Int. | H&N | Brain injury; Cognitive function | Predictive | MRI |
| ElNaqa 2018[90] | N. America | Clinical (RWD) | Retrosp. | Long. | No | None | GI | Liver injury | Prognostic | MRI |
| Fahlstrom 2018[91] | Europe | Clinical (RWD) | Prosp. | Long. | No | None | Neuro. | Brain injury | Diagnostic; Predictive | MRI |
| Lv 2019[92] | Asia-Pacific | Clinical (RWD) | Prosp. | Long. | No | None | H&N | Brain injury; Cognitive function | Diagnostic; Predictive | MRI |
| Meheissen 2019[93] | N. America | Clinical (RWD) | Prosp. | Long. | No | None | H&N | Dysphagia | Monitoring; Predictive | MRI |
| vanDijk 2018[94] | N. America | Clinical (RWD) | Retrosp. | Cross sect. | No | Int. & Ext. | H&N | Xerostomia | Prognostic | MRI |
| Veiga 2018[95] | Europe | CT | Prosp. | Long. | No | None | Thoracic | Lung injury | Safety | 4DCT; CT; PET-CT |
| Winter 2018[96] | N. America | CT | Prosp. | Long. | No | None | Neuro. | Brain necrosis | Predictive; Pharmacodynamics or response | MRI |
| Zhang 2018[97] | Asia-Pacific | Clinical (RWD) | Prosp. | Cross sect. | No | None | H&N | Encephalopathy | Diagnostic; Monitoring | MRI |
| Kundu 2017[98] | N. America | Clinical (RWD) | Retrosp. | Long. | No | None | Neuro. | Brain injury | Monitoring | MRI |
| Ma 2017[99] | Asia-Pacific | Clinical (RWD) | Prosp. | Cross sect. | No | Int. | H&N | Cognitive function | Monitoring | MRI |
| Molad 2017[100] | Middle East | CT | Prosp. | Long. | No | Int. | Neuro. | Brain injury | Susceptibility or risk | MRI |
| Niedzielski 2017[101] | N. America | CT | Retrosp. | Long. | No | None | Thoracic | Esophagitis | Safety | 4DCT |
| Niedzielski 2017[102] | N. America | Clinical (RWD) | Prosp. | Long. | No | None | Thoracic | Esophagitis | Predictive | 4DCT |
| vanDijk 2017[103] | Europe | Clinical (RWD) | Prosp. | Long. | No | Int. | H&N | Xerostomia | Predictive | CT |

| Study ID | Region | Type | Timing | Temp. | Clin. Impl. | Study Val. | Cancer | Clin. Endpoint | BEST Cat. | Imaging |
|---|---|---|---|---|---|---|---|---|---|---|
| Xie 2018[104] | Asia-Pacific | Clinical (RWD) | Retrosp. | Cross sect. | No | None | H&N | Brain injury | Diagnostic | MRI |
| Yoon 2017[105] | Asia-Pacific | Clinical (RWD) | Retrosp. | Cross sect. | No | Int. | Neuro. | Brain necrosis | Diagnostic | MRI |
| Ahmad 2016[106] | N. America | Clinical (RWD) | Retrosp. | Long. | No | None | H&N | Brain necrosis | Diagnostic; Monitoring | MRI |
| Farjam 2015[107] | N. America | Clinical (RWD) | Prosp. | Long. | No | None | Neuro. | Brain injury | Predictive | MRI |
| Meheissen 2016[108] | N. America | Clinical (RWD) | Prosp. | Long. | No | None | H&N | ORN | Diagnostic; Monitoring | MRI |
| Ma 2016[109] | Asia-Pacific | Clinical (RWD) | Retrosp. | Cross sect. | No | Int. | H&N | Cognitive function | Monitoring | MRI |
| Seibert 2017[110] | N. America | Clinical (RWD) | Retrosp. | Long. | No | None | Neuro. | Brain injury | Monitoring | MRI |
| Wong 2016[111] | N. America | Clinical (RWD) | Prosp. | Long. | No | None | Neuro. | Brain injury | Monitoring | MRI |
| Castillo 2015[112] | N. America | Clinical (RWD) | Retrosp. | Cross sect. | No | Int. | GI | Pneumonitis | Prognostic | PET-CT |
| Li 2015[113] | Asia-Pacific | Clinical (RWD) | Retrosp. | Cross sect. | No | None | Neuro. | Brain injury | Diagnostic | MRI |
| Castillo 2014[114] | N. America | Clinical (RWD) | Retrosp. | Long. | No | Int. | Thoracic | Pneumonitis | Prognostic | PET-CT |
| Hsieh 2014[115] | Asia-Pacific | Clinical (RWD) | Retrosp. | Long. | No | Int. | H&N | Trismus | Predictive; Prognostic | MRI |
| Nazem-Zadeh 2014[116] | N. America | Clinical (RWD) | Prosp. | Long. | No | Int. | Neuro. | Brain necrosis | Predictive | MRI;<br>PET-MR |
| Yang 2014[117] | N. America | Clinical (RWD) | Prosp. | Long. | No | Int. | H&N | Xerostomia | Diagnostic; Monitoring | US |
| Abdulla 2014[118] | N. America | Clinical (RWD) | Retrosp. | Long. | No | None | Thoracic | Pneumonitis | Monitoring | PET-CT |
| Chung 2013[119] | Asia-Pacific | Clinical (RWD) | Retrosp. | Long. | No | Int. | Neuro. | Brain necrosis | Diagnostic | MRI |
| Hope 2015[120] | Europe | Clinical (RWD) | Prosp. | Long. | No | None | Neuro. | Brain injury | Predictive | MRI |
| Lam 2013[121] | N. America | CT | Retrosp. | Cross sect. | No | None | GI | Liver injury | Predictive; Prognostic | SPECT/CT |
| Martens 2014[122] | Europe | Clinical (RWD) | Prosp. | Cross sect. | No | None | GI | Fibrosis | Diagnostic | MRI |
| Ravn 2013[123] | Europe | Clinical (RWD) | Retrosp. | Cross sect. | No | None | Neuro. | Brain injury | Predictive | MRI |
| Chapman 2012[124] | N. America | Clinical (RWD) | Prosp. | Long. | No | Other | Neuro. | Brain injury | Predictive | MRI |
| Sanuki 2012[125] | Asia-Pacific | Clinical (RWD) | Retrosp. | Long. | No | Int. | Thoracic | Pneumonitis | Predictive | CT;<br>X-ray |
| Cannon 2012[126] | N. America | Clinical (RWD) | Prosp. | Long. | No | Prosp. | H&N | Xerostomia | Predictive | PET-CT |
| D'Errico 2012[127] | Europe | Clinical (RWD) | Prosp. | Long. | No | None | Breast | Cardiac injury | Predictive | CT;<br>ECG |
| Narang 2011[128] | N. America | Clinical (RWD) | Retrosp. | Cross sect. | No | Int. | Neuro. | Brain necrosis | Diagnostic | MRI |
| Yamashita 2010[129] | Asia-Pacific | Clinical (RWD) | Retrosp. | Long. | No | Int. | Thoracic | Pneumonitis | Predictive | CT |
| Cao 2009[130] | N. America | Clinical (RWD) | Prosp. | Long. | No | Int. | Neuro. | Brain injury; Cognitive | Predictive | MRI |

| Study ID | Region | Type | Timing | Temp. | Clin. Impl. | Study Val. | Cancer | Clin. Endpoint | BEST Cat. | Imaging |
|---|---|---|---|---|---|---|---|---|---|---|
| | | | | | | | | function | | |
| Sundgren 2009[131] | N. America | Clinical (RWD) | Retrosp. | Long. | No | None | Neuro. | Cognitive function | Predictive | MRI |
| Nagesh 2008[132] | N. America | Clinical (RWD) | Prosp. | Long. | No | None | Neuro. | Brain injury | Safety | MRI |

N. America=North America

Clinical (RWD)=Clinical (Real-world data); CT=Clinical trial

Prosp.=Prospective; Retrosp.=Retrospective

Cross sect.=Cross sectional; Long.=Longitudinal

GI=Digestive/Gastrointestinal; H&N=Head and neck; Neuro.=Neurologic; Thoracic=Respiratory/Thoracic

ORN=Osteoradionecrosis

4DCT=four-dimensional CT; CBCT=Cone-beam CT; CT=Computed tomography; ECG=Electrocardiogram; MRI=Magnetic resonance imaging; PET-CT=Positro
SPECT/CT=single-photon emission CT/CT; US=Ultrasound

### 3.2.2 Study Design and Data

86% of studies were classified as a clinical study using real-world data, while 14% were clinical trials. 51% of studies used a prospective analysis, while 49% used retrospective. 91 (89%) were cohort studies, followed by randomized controlled trials (8) and case control studies (8), and 1 non-randomized experimental study. 31% were cross-sectional studies, while 69% were longitudinal. The study population size distribution was very similar for clinical studies (median 59, IQR: 26-116.5) and clinical trials (median 59, IQR: 26.5–116). The median population size for prospective studies was 47 (IQR: 25-89.5), while the median population size for retrospective studies was 87 (IQR: 34-154.25).

Intensity-modulated radiation therapy (IMRT) was used most often in the studied cohorts; full treatment modality distribution is shown in Supplementary Figure S1. 62% of studies used radical treatment methods, followed by 21% adjuvant, 4% neoadjuvant, 2% palliative, and 1% re-irradiation (10% not specified). 56% of studies had IBM integration with dosimetric data; of these studies, 64% investigated predictive biomarkers.

MRI was the most common imaging modality used (67.6%), followed by CT (19.6%) and PET-CT (12.7%). The full distribution of imaging modalities is shown in Supplementary Figure S2.

The most common cancer types studied were neurologic (36, 35%) and head and neck (33, 32%), followed by respiratory/thoracic (17, 17%), digestive/gastrointestinal (11, 11%), breast (3, 3%) and genitourinary (2, 2%). Cancer type stratified by gender is shown in Fig. 3.

Brain injury, brain necrosis, and cognitive function were studied most frequently; more detailed results on these are provided in section 3.2.3 below. All clinical endpoints assessed are shown in Supplementary Figure S3 with cancer type stratification shown in Fig. 4. Other frequently studied endpoints included radiation-induced xerostomia, radiation pneumonitis, dysphagia, and cardiac injury.

With regards to BEST biomarker category, predictive biomarkers were assessed the most at 41% of studies (Supplementary Figure S4), particularly for head and neck and neurological cancers (Fig. 5). No studies involved clinical implementation or regulatory approval.

Study validation was reported in 51% of studies (40% internal, 6% external, 3% prospective, 2% other), whereas technical validation of imaging biomarkers was reported in 43% of studies. 34% of studies employed machine learning and/or deep learning methods. 60% of studies involving prediction model development assessed their model performance; the most common methods used were classified as discrimination and calibration.

### 3.2.3 Neurological Cancers

The most common neurological endpoints in this review were brain necrosis and cognitive function. Brain necrosis was addressed in 11 studies spanning glioblastoma, high-grade glioma, and brain metastases[35,41,42,45,65,71,96,105,116,119,128]. The central clinical challenge was distinguishing radiation-induced necrosis from tumor recurrence on conventional imaging, which drove the predominance of diagnostic biomarker BEST classifications[35,41,42,65,71,105,119,128]; a smaller subset evaluated predictive biomarkers to stratify necrosis risk[45,96,116], with select studies carrying additional prognostic[42,65,71] or pharmacodynamics/response[96] classifications. MRI dominated imaging approaches, with advanced functional MRI sequences outperforming conventional imaging across diagnostic studies. Three studies integrated dosimetric data: Winter et al.[96] correlated ADC and Ktrans with prescription dose; Nazem-Zadeh et al.[116] combined diffusivity features with dose-volume parameters; and Alirezaei et al.[45] incorporated dosimetric inputs in an NTCP-based predictive model. Reported diagnostic AUCs ranged from 0.73 for single diffusion parameters to 0.95 for multiparametric approaches[105], and predictive models showed sensitivity/specificity of 83–89%/81–86%[45,116], but all evidence came from small retrospective cohorts, and no biomarker was prospectively validated. Cognitive function was addressed in seven studies of primary brain tumor radiotherapy[38,40,59,81,87,130,131]. All used MRI, targeting hippocampal, medial temporal lobe, and corpus callosum structures; tumor types spanned low-grade glioma, high-grade glioma, glioblastoma, and metastatic medulloblastoma. Predictive biomarkers dominated and showed correlation between MRI features with radiation-induced cognitive decline as measured with cognitive test scores. Four of seven studies[59,81,130,131] demonstrated dose-dependent changes in imaging parameters. Piffer et al.[40] combined dosiomic and radiomic features into a unified predictive signature for neurotoxicity stratification.

#### 3.2.4 Head and Neck Cancers

In papers regarding head and neck cancers, xerostomia and dysphagia were among the most notable clinical endpoints assessed. Papers developed BEST biomarkers of xerostomia, including monitoring[117], pharmacodynamics or response[69], predictive[50,52,63,69,78,103,126], and prognostic[52,94] categories. CT[52,69,103], MRI[69,94], and ultrasound[117] were commonly used to assess xerostomia, although PET-CT[50,63,78,126] encompassed the majority of predictive studies. For xerostomia after head and neck RT, several imaging features improved prediction over dose-based models: parotid CT texture[106], MRI fat-related intensity[94], FDG-PET uptake[50,78], and a deep-learning model on parotid CT images in oropharyngeal cancer[52]. Changes measured during treatment were associated with side effects for FDG-PET uptake of the parotid glands and xerostomia[50] and T2 signal of the swallowing muscles and moderate-to-severe dysphagia in HPV-positive oropharyngeal cancer[93]. MRI was the sole imaging modality to assess the predictive biomarker capacity of dysphagia[39,93]. Six head and neck studies reported AUC values for prediction with the best models achieving results 0.77–0.85, but most of these were developed from small, internal cohorts (< 108 patients) [50,52,63,69,78,103]. The outcome assessment was also found to be varied amongst these studies; six studies used various clinician grading tools[50,52,63,69,117,126], while van Dijk et al.[103] and Wilkie et al.[78] used PROMs.

### 4. Discussion

This scoping review characterizes the current landscape of imaging biomarkers for the detection, monitoring, and prediction of radiation-induced side effects across cancer types and treatment modalities. Despite a growing body of literature, imaging biomarkers for radiotherapy-related normal tissue damage remain predominantly investigational. No studies reported clinical implementation or regulatory approval, underscoring a clear translational gap between biomarker discovery and routine clinical use.

The methodological shortcomings identified in this review are best understood in the context of established frameworks for imaging biomarker development and translation. O'Connor et al.[25] outlined a structured imaging biomarker roadmap for cancer studies that defines a staged translational pathway including technical validation, biological validation, clinical validation, demonstration of clinical utility, and regulatory approval before a biomarker can be considered ready for routine practice. When mapped against this roadmap, the majority of imaging biomarkers in our review remain at the earliest stages. 57% of studies did not technically validate their biomarkers, and external validation was present in only 6% of included studies[32,47,62,78,94]. This pattern indicates that biomarker research frequently falls short of the prospective, multi-center validation studies required for clinical translation.

With regards to the added value of imaging beyond clinical and dosimetric variables, there are a number of ways that studies integrated IBM with clinical/dosimetric data: 1) twenty formally compared a model built with imaging-derived features against a purely clinical and/or dosimetric one; 2) fifteen analyzed imaging together with dose-related or clinical variables but never reported how those variables performed on their own; and 3) eleven used imaging alone to distinguish RT-induced tissue damage from tumor, with no clinical or dosimetric variables at all. Despite this integration, few studies did a formal comparison with and without clinical/dosimetric data; thus, it is difficult to conclude if this adds sufficient value. Neurological cancers offer one example where imaging did appear to add value: where cognitive outcomes were assessed, imaging features predicted these more reliably than dosimetric variables alone, suggesting that imaging may capture individual radiation sensitivity not reflected by dose-volume metrics. However, these conclusions were developed off of small, retrospective, single-center cohorts and had no external validation. Only six of the twenty comparative studies validated their model in a different institution or an independent cohort[33,47,62,72,94,117], and with the exception of the study by Liu et al.[48], those validation sets were small with roughly 20 to 70 patients. The rest relied on internal validation, or none at all.

Overall, imaging changes during or shortly after RT are promising but rest on small, mostly single-center series, and no studies that compared them with a clinical model were validated externally. The clearest example came from neurological cancers, where structural and metabolic MRI biomarkers detected radiation-induced brain injury earlier and with greater sensitivity than standardized cognitive testing, with changes evident in normal-appearing tissue weeks to months before cognitive test deterioration. Direct comparison of longitudinal changes with baseline-only or single-scan features was rare, and gains were modest or not significant[50,68,69,76]. Signals varied with the metric and the acquisition window, and no study used an imaging change to actually adapt treatment. The incremental value of longitudinal imaging over a single baseline scan therefore remains largely untested.

Technical standardization initiatives such as QIBA[133], which focuses on reducing variability in image acquisition protocols across devices and sites, and IBSI, which targets the computational extraction of quantitative image features by standardizing feature definitions, preprocessing workflows, and software implementations, address complementary aspects of the IBM pipeline. The diversity of imaging modalities observed across studies in this review mostly reflects the clinical imaging infrastructure available at each institution and is not, in itself, a marker of poor standards uptake. Nevertheless, prospective IBM studies, especially multi-center studies where harmonized data collection across sites is essential, would benefit from prospective alignment with QIBA profiles from the outset to ensure that biomarker measurements are comparable across institutions and time points. The heterogeneity in analytical pipelines represents a more avoidable source of variability, and one that IBSI was specifically designed to address. The extent to which included studies adopted IBSI-compliant extraction workflows was not systematically reported, but the absence of such reporting is itself an indicator of inconsistent engagement with available standardization tools.

Similarly, reporting frameworks such as the Radiomics Quality Score (RQS)[18], TRIPOD(+ AI)[19], STARD[20], CLAIM[21], and REMARK[22] have been developed precisely to improve transparency, reproducibility, and rigour in biomarker studies. Notably, no studies in our review included an EQUATOR reporting checklist, despite the fact that several of these guidelines, including REMARK (2005), STARD (2015), and TRIPOD (2015), were available to the vast majority of included studies at the time of publication. Even RQS, introduced in 2017, predates the bulk of the literature reviewed here, given that 82% of studies were published between 2016 and 2024. This suggests that non-adherence reflects not a lack of available guidance, but insufficient uptake.

Based on the FDA-NIH BEST framework[26], our review found that predictive biomarkers were the most commonly studied category (53% of studies), particularly in neurological cancer (18) and head and neck cancer (18). It is worth acknowledging that classifying studies according to the BEST framework was not always straightforward in practice, as the boundaries between predictive, monitoring, and diagnostic biomarker categories were frequently blurred in the included literature. Beyond this classification challenge, a broader conceptual point deserves consideration: all imaging biomarkers evaluated in this review could, regardless of their assigned BEST category, be considered safety biomarkers. Within the BEST taxonomy, a safety biomarker indicates the presence, severity, or likelihood of an adverse effect from a therapeutic exposure[26]. Since every IBM in this review was developed to detect, monitor, or predict radiation-induced normal tissue injury, each is by definition a marker of treatment-related harm. Explicitly framing IBM for RT-related side effects as safety biomarkers has practical implications: safety biomarkers carry specific regulatory expectations regarding technical performance and clinical utility, and their formal qualification through pathways such as the FDA Biomarker Qualification Program or equivalent European mechanisms may be more accessible than has been recognized. Future studies should engage with this classification deliberately when designing validation frameworks and planning regulatory strategies.

Additionally, there was a scarcity of studies specifically addressing longitudinal monitoring and studies exploring regulatory-focused safety biomarker development, which further highlights how incompletely the field has engaged with the full translational pathway envisioned by these frameworks.

Several methodological patterns in the included studies warrant specific attention when considering future research priorities. The predominance of single-institution studies (85%) and small cohort sizes, with median population sizes of approximately 59 patients across both clinical studies and clinical trials, represents a persistent structural limitation. Small, single-center studies are highly susceptible to overfitting, selection bias, and the influence of institution-specific imaging protocols, all of which compromise the generalizability and reproducibility of derived IBM. One methodologically distinct response to this challenge is illustrated by Sadique et al. (2024)[41], the only in silico study in this review, which employed synthetic data generation via generative models to address class imbalance arising from the scarcity of radiation necrosis patient data. While this computational approach is not a substitute for real-world clinical validation, it highlights a potentially valuable strategy for augmenting limited datasets in rare or underrepresented side effect phenotypes. Ultimately, however, prospective multi-center studies, ideally embedded within existing clinical trial infrastructure, are needed to generate the volume and diversity of data required for robust IBM development and external validation.

The integration of IBM with dosimetric data remains underdeveloped. While dose-volume relationships are central to understanding and predicting radiation-induced side effects, the degree to which included studies systematically incorporated dosimetric variables alongside imaging biomarkers was limited; however, in the majority of these studies the dosimetric information was used for prediction of side effects (i.e., BEST predictive IBM). Future RT-related IBM studies should be designed from the outset to capture and report dosimetric parameters alongside imaging data, enabling the development of integrated predictive models that reflect the underlying radiobiological mechanisms of tissue injury.

The growing application of machine learning and deep learning methods, present in 34% of included studies, reflects a broader trend in medical imaging research and offers analytical potential. However, the limited use of external validation suggests that many ML/DL-based image biomarkers require considerably more rigorous evaluation before clinical deployment. The adoption of frameworks such as TRIPOD+AI[19] and CLAIM[21] should be considered for studies employing these methods.

It is worth mentioning that the pilot MANTLE trial[134] represents a notable methodological departure from the observational majority of included studies. This NCI-funded, prospectively registered single-arm trial evaluated manual therapy for fibrosis-related late-effect dysphagia in head and neck cancer survivors following radiotherapy, with quantitative MRI, including longitudinal T1 signal intensity as a candidate biomarker of radiation-associated soft tissue change, incorporated as an exploratory endpoint alongside functional and patient-reported outcomes. What distinguishes MANTLE within this review is the fact that the image biomarker was embedded within an interventional framework designed to test whether a clinical intervention changes measurable tissue and functional outcomes, rather than serving as the primary study objective. This represents the kind of trial architecture needed to ultimately demonstrate clinical utility, i.e., the highest bar in the O'Connor translational roadmap, and stands as a model for how imaging biomarkers might be more purposefully integrated into future interventional studies of RT-related side effects.

This review has several limitations that should be considered when interpreting its findings. First, as a scoping review, it was designed to map the breadth of the existing literature rather than to perform a formal quality assessment or meta-analysis. Accordingly, no pooled quantitative synthesis of IBM performance was conducted, and the heterogeneity of outcome measures, imaging modalities, and cancer types across studies precluded direct comparisons. Second, the review was limited to studies indexed in MEDLINE, Embase, and the Cochrane Library up to January 2025; studies published after this date, or those appearing only in grey literature or conference proceedings, were not captured. Third, we acknowledge that while multiple databases were searched, there is no guarantee that all relevant articles were captured in this scoping review. While a manual search was not included in the search methodology, this scoping review still encompassed a good representation of the studies in the field. Additionally, we acknowledge that articles may have been published after analysis, as this is a very active and evolving field. Finally, while data extraction was performed independently by at least two reviewers and disagreements were resolved by consensus, some degree of subjectivity in the classification of IBM categories, validation approaches, and clinical implementation status is inherent in a review of this scope.

### Declarations

// Conflicts of Interest

## Conflicts of Interest

CDF has received unrelated travel/honoraria/speaking fees from Elekta AB, Philips Medical Systems, Siemens Healthineers/Varian, and GE Healthcare. CDF receives unrelated royalties via the UT System from Kallisio, Inc. CDF receives unrelated salary support as an NIH sub-awardee through NRG Oncology. LHV has received funding from the European Union's Horizon Europe research and innovation programme under the Marie Sklodowska-Curie grant agreement No 101179122.

**Competing Interests**

NAW is supported by a training fellowship from UTHealth Houston Center for Clinical and Translational Sciences T32 Program (Grant No. T32 TR004905), the American Legion Auxiliary Fellowship in Cancer Research, and the Ray Meyn Scholarship for Cancer Research. PvL is supported by grants 11349 and 16197 of the Dutch Cancer Society. CF receives grant support from AIRC (IG 2025 ID 32254); project "TETRIS" HORIZON-EURATOM-2023-NRT-01 Grant number 101166699. CDF has received directly related funding and program support from the National Institutes of Health (NIH) National Institute of Dental and Craniofacial Research (NIDCR; R01DE028290), and the NIH National Cancer Institute (NCI) MD Anderson Cancer Center Support Grant (CCSG) Program (P30CA016672). CDF has received unrelated travel/honoraria/speaking fees from Elekta AB, Philips Medical Systems, Siemens Healthineers/Varian, and GE Healthcare. CDF receives unrelated royalties via the UT System from Kallisio, Inc. CDF receives unrelated salary support as an NIH sub-awardee through NRG Oncology. LHV has received funding from the European Union's Horizon Europe research and innovation programme under the Marie Sklodowska-Curie grant agreement No 101179122.

## Funding Declaration

NAW is supported by a training fellowship from UTHealth Houston Center for Clinical and Translational Sciences T32 Program (Grant No. T32 TR004905), the American Legion Auxiliary Fellowship in Cancer Research, and the Ray Meyn Scholarship for Cancer Research. PvL is supported by grants 11349 and 16197 of the Dutch Cancer Society. CF receives grant support from AIRC (IG 2025 ID 32254); project "TETRIS" HORIZON-EURATOM-2023-NRT-01 Grant number 101166699. CDF has received directly related funding and program support from the National Institutes of Health (NIH) National Institute of Dental and Craniofacial Research (NIDCR; R01DE028290), and the NIH National Cancer Institute (NCI) MD Anderson Cancer Center Support Grant (CCSG) Program (P30CA016672).

## CRediT Author Statement

Conceptualization: NAW, CF, RJ, TR, CDF, LHV. Methodology: NAW, DPF, LHV. Software: LHV. Validation: NAW, LHV. Formal analysis: NAW, LHV. Investigation: NAW, EG, AA, JS, GRG, VP, PvL, LHV. Resources: DPF, LHV. Data Curation: NAW, LHV. Writing - Original Draft: NAW, LHV. Writing - Review & Editing: NAW, EG, AA, JS, GRG, VP, DPF, PvL, CF, RJ, TR, CDF, LHV. Visualization: NAW, LHV. Supervision: LHV. Project Administration: LHV. Funding Acquisition: NAW, CDF, LHV.

## Data Availability

All data supporting the findings of this study are available within the paper and its Supplementary Information.

### Figures

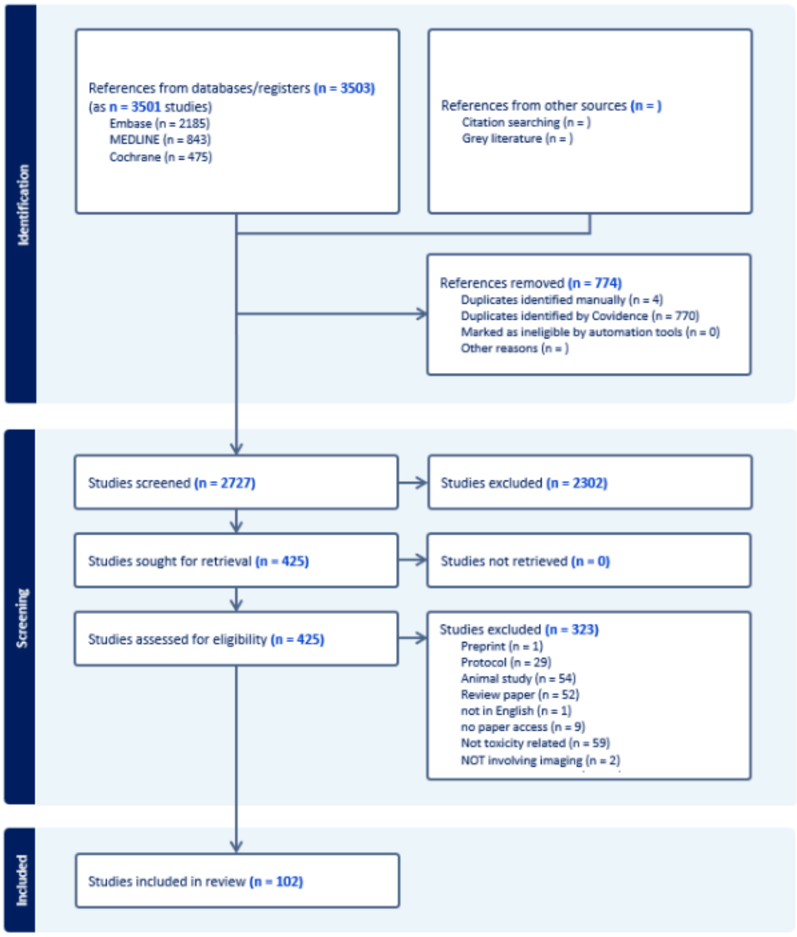

Figure 1

**PRISMA-ScR flow diagram of study selection.** Records were identified from three databases (n = 3,503) and screened following deduplication (n = 2,727). After title/abstract and full-text review, 102 studies were included in the final analysis.

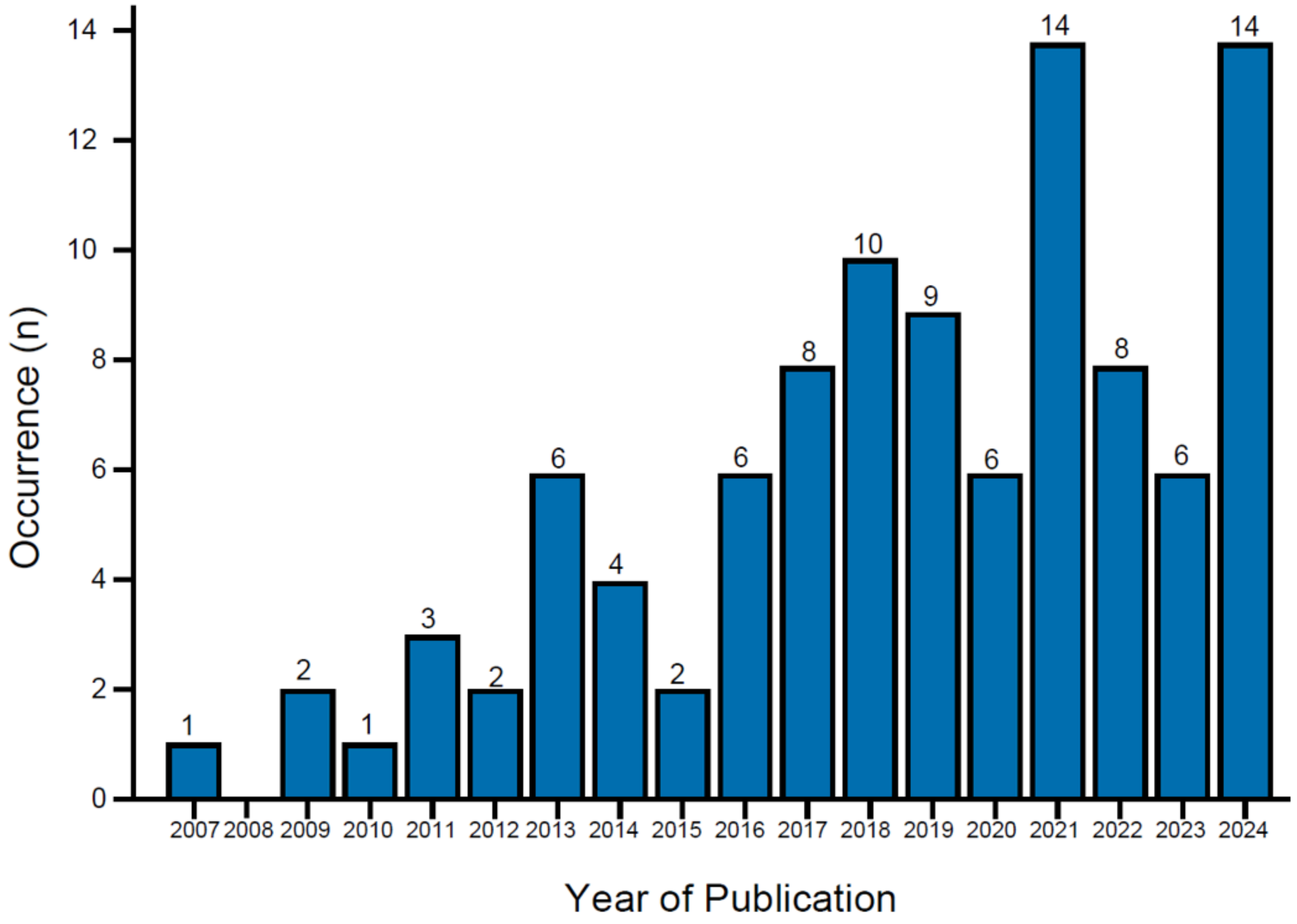

**Figure 2**

**Distribution of included studies by year of publication (2007-2024).** Publication volume increased substantially from 2016 onwards, with 82% of studies published between 2016 and 2024.

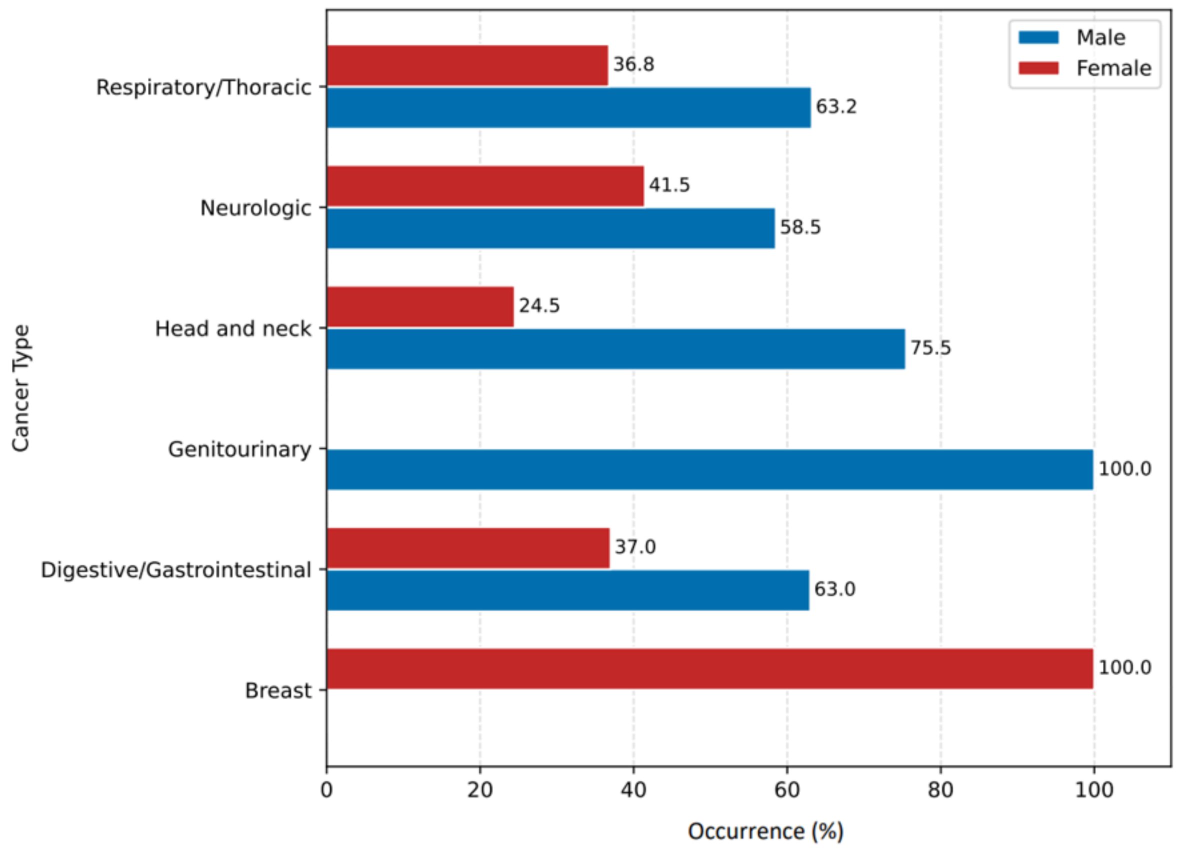

Figure 3

**Distribution of cancer types studied, stratified by gender.** Horizontal bar charts show the median proportion of male (blue) and female (red) patients across studies for each cancer type.

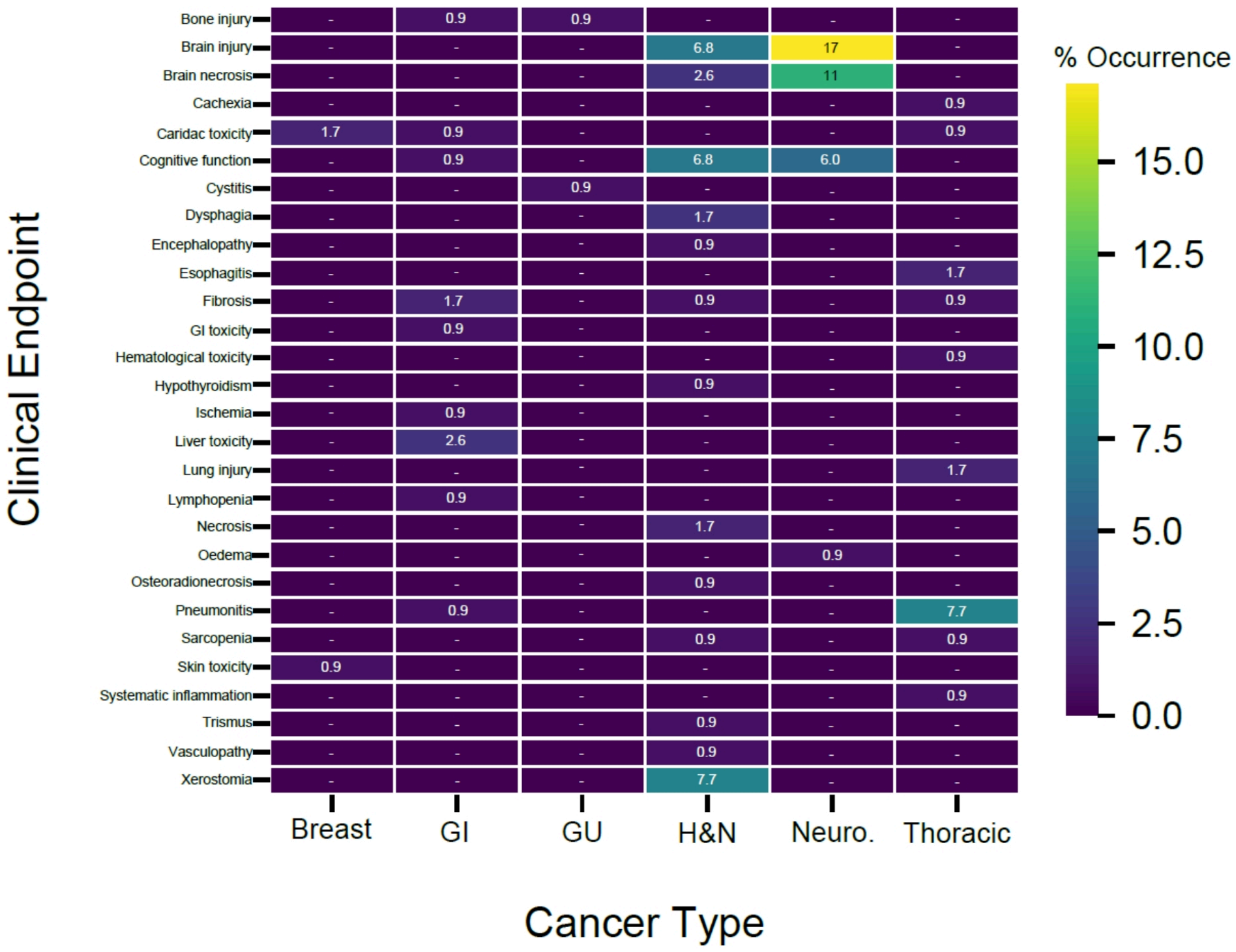

Figure 4

**Distribution of clinical endpoints assessed, stratified by cancer type.** Heatmap showing the proportion of studies per clinical endpoint and cancer type. “-” indicates no cases of the combined cancer type and clinical endpoint.

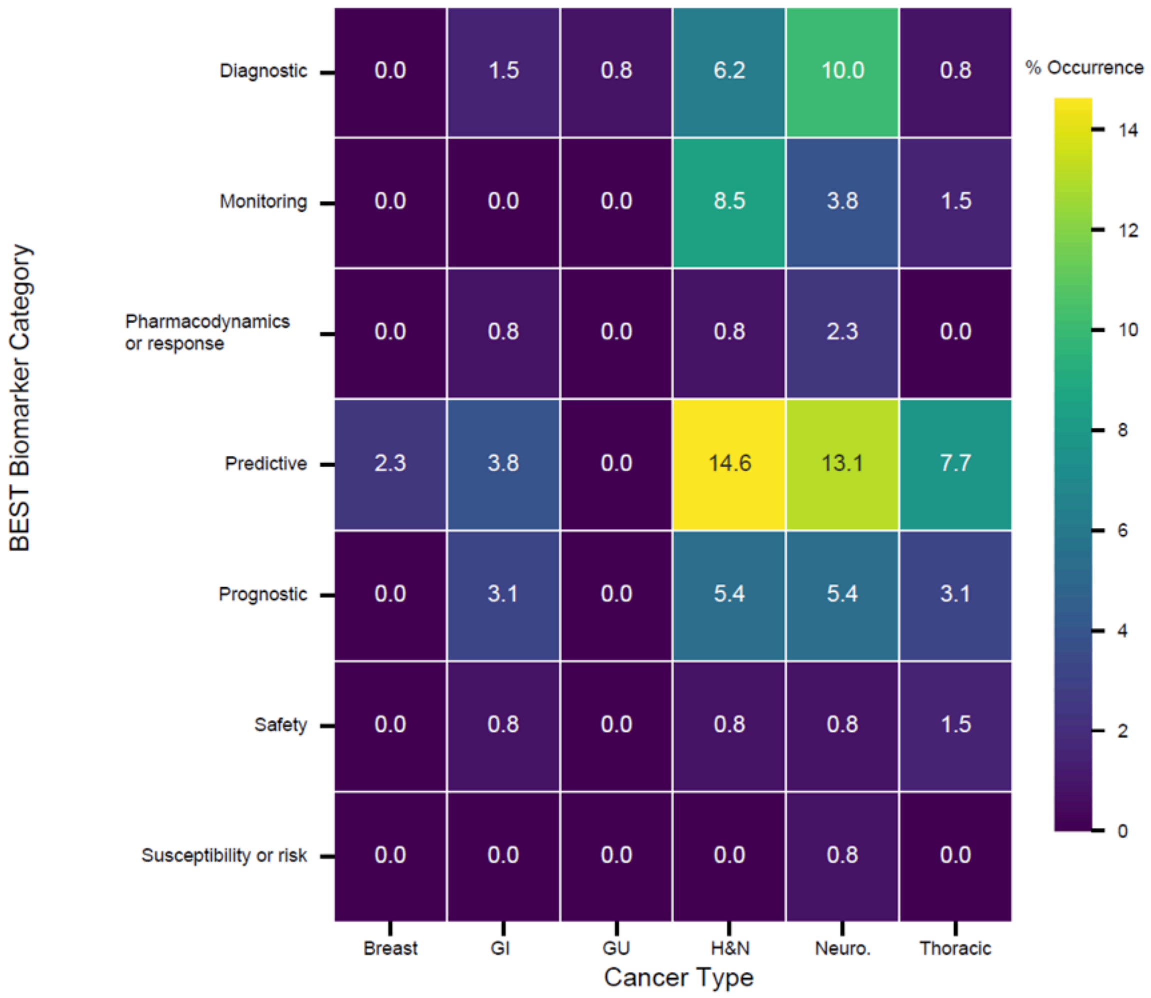

Figure 5

**Distribution of FDA-NIH BEST biomarker categories[26] across cancer types.** Heatmap displaying the percent of studies per BEST category and cancer type, showing the highest occurrence for predictive biomarkers in head and neck cancer and neurological cancers.

### Supplementary Files

This is a list of supplementary files associated with this preprint. Click to download.

- SupplementaryMaterialforreview.docx
